# Clinical significance of serum and urinary HER2/neu protein levels in primary non-muscle invasive bladder cancer

**DOI:** 10.1590/S1677-5538.IBJU.2014.0628

**Published:** 2015

**Authors:** Ozgur Arikan, Asýf Yýldýrým, Banu Ýsbilen, Cengiz Canakci, Gokhan Atýs, Cenk Gurbuz, Bulent Erol, Ferruh Kemal Ýsman, Seyma Ozkanli, Turhan Caskurlu

**Affiliations:** 1 Department of Urology, Nigde Bor State Hospital, Bor Nigde, Ýstanbul, Turkey; 2 Department of Urology, Istanbul Medeniyet University Goztepe Research and Training Hospital, Istanbul, Turkey; 3 Department of Biochemistry, Istanbul Medeniyet University Goztepe Research and Training Hospital, Istanbul, Turkey; 4 Department of Pathology, Istanbul Medeniyet University Goztepe Research and Training Hospital, Istanbul, Turkey

**Keywords:** HER2-neu-derived peptide (654-662) [Supplementary Concept], Serum, Urinary Bladder Neoplasms

## Abstract

**Objective:**

We aimed to compare serum and urinary HER2/neu levels between healthy control group and patients with non-muscle invasive bladder cancer. Additionally, we evaluated relationship of HER2/neu levels with tumor stage, grade, recurrence and progression.

**Materials and Methods:**

Fourty-four patients with primary non-muscle invasive bladder tumors (Group 2) and 40 healthy control group (Group 1) were included the study. Blood and urinary samples were collected from all patients and HER2/neu levels were measured by ELISA method. Blood and urinary HER2/neu levels and additionally, ratio of urinary HER2/neu levels to urinary creatinine levels were recorded. Demographic data and tumor characteristics were recorded.

**Results:**

Mean serum HER2/neu levels were similar between two groups and statistically significant difference wasn't observed. Urinary HER2/neu levels were significantly higher in group 2 than group 1. Ratio of urinary HER2/neu to urinary creatinine was significantly higher in group 2 than group 1, (p=0,021). Serum and urinary HER2/ neu levels were not associated with tumor stage, grade, recurrence and progression while ratio of urinary HER2/neu to urinary creatinin levels were significantly higher in high-grade tumors. HER2/neu, the sensitivity of the test was found to be 20.5%, and the specificity was 97.5%, also for the urinary HER2/neu/urinary creatinine ratio, the sensitivity and specificity of the test were found to be 31.8% and 87.5%, respectively.

**Conclusions:**

Urinary HER2/neu and ratio of urinary creatinine urine were significantly higher in patients with bladder cancer compared to healthy subjects. Large series and controlled studies are needed for use as a tumor marker.

## INTRODUCTION

Bladder cancer is the second most frequently diagnosed genitourinary malignancy after prostate cancer. Approximately 70,000 new cases of bladder cancer were detected in the United States of America (USA) in 2007, and bladder cancer is estimated to occur in 500,000 people (1, 2). The most common histological type of bladder cancer is transitional cell carcinoma, which is responsible for up to 95% of all bladder cancers (3).

Because of the invasive and uncomfortable nature of cystoscopy, new methods are being investigated for diagnosing bladder cancer and identifying the risks of recurrence and progression. Urinary cytology is a non-invasive method that has been used for many years. The sensitivity of urinary cytology is poor (20-60%), however, its specificity, which is dependent on the tumor grade, surpasses 90%, particularly in high-grade tumors (4–6). The low sensitivity and grade-dependent specificity of urinary cytology as well as the significant role of the pathologist's experience in determining accuracy have necessitated research for new tumor markers to diagnose bladder cancer. Because tumor suppressor genes and oncogenes have been reported to be associated with bladder cancer and may have the potential for developments in technology and molecular biology, these new molecules are perceived to contain tumor markers for bladder cancer.

The HER2/neu (C-erbB-2) is a member of the epidermal growth factor receptor (EGFR) family and is responsible for cell growth and proliferation by activating the tyrosine kinase pathway. HER2/neu (C-erbB-2) has been shown to exhibit over-expression in tumor tissues of breast, colon, gastric, lung and bladder cancer (7–11). The expression of HER2/neu can be determined using different methods, such as immunohistochemistry (IHC), enzyme-linked immunosorbent assay (ELISA), fluorescent in situ hybridization (FISH), and polymerase chain reaction (PCR) (12). Many studies have investigated HER2/neu expression in tumor tissues in patients with bladder cancer. However, according to the literature review conducted in the present study, only one study has evaluated the HER2/neu protein level in serum and urinary samples (13).

The present study aimed to compare the HER2/neu protein levels in serum and urine samples of the patients with non-muscle invasive bladder cancer (NMIBC) and the same levels in the healthy control subjects in similar age groups.

Urinary HER2/neu concentrations were normalized to the concentrations of urinary creatinine because of the concentration of the urine itself may affect the interpretation of a urinary biomarker and to reduce the variations due to dilution (14). Additionally, we aimed to analyze the correlation of HER2/neu protein levels with the stage, grade, recurrence and progression of tumors.

## MATERIALS AND METHODS

The present study was performed in accordance with the Declaration of Helsinki between September 2012 and October 2013, with the approval (28.08.2012-25/P) of the Local Ethics Committee. A total of 84 patients (n=62 men, 22 women) were included in the study. Group-1 (control group) was composed of 40 healthy volunteers (n=25 men, 15 women) and Group-2 was composed of 44 patients (n=37 men, 7 women), who were diagnosed with NMIBC (Ta, T1-WHO 2009). During the collection of urinary and serum samples, patients with infection, macroscopic hematuria, radiologically evident invasive bladder cancer and known patients with other primary malignancy were excluded from the study. The demographic data, body-mass index (BMI), and history of smoking were recorded for all of the patients. The tumor stage (Ta and T1 using UICC 2002 TNM staging system), grade (low-or high-grade according to the WHO 2004 grading system), presence of carcinoma in situ (CIS), size and number of tumors, and recurrence and progression of tumors were recorded in a follow-up period for the patients with bladder cancer. Before surgery, the patients with bladder cancer were submitted to the NMP22 Bladder Check test (Matritech Inc., Newton, MA, USA) and urinary cytology. NMP22 test results were obtained according to the manual's guidelines, four drops of urine were dropped on the kit, and we observed and recorded the changes after a 30 minute wait. A color band in the test position indicates a positive result. Fresh urine samples were collected from the patients using sterile containers for cytology, the samples were examined by a pathologist who had no prior knowledge of the patient group. In the cytology examination, the presence of atypical and malignant cells was considered as malignant cytology, whereas the absence of these types of cells was considered as benign cytology. After surgery, the patients were administered intravesical treatment quantitative data were analyzed using the chi-squared test, and when the conditions of the chi-squared test were not fulfilled, the analyses were conducted using Fisher's exact test. The correlation was checked using Spearman's correlation analysis. The receiver operating characteristics (ROC) curve was analyzed to calculate the predictive values, whereas the Kappa statistic was used to determine sensitivity and specificity. A p<0.05 was considered to be statistically significant. The analyses were conducted using the SPSS 21.0 (SPSS Inc., Chicago, IL, USA).

## RESULTS

The mean ages of the patients were 62±9.6 years (range, 34-80 years) in Group-1 and 63.9±11.1 years (range, 43-84 years) in Group-2 (p=0.488). Group-1 was composed of 15 females (37.5%) and 25 males (62.5%), and Group-2 was composed of 14 females (31.8%) and 30 males (68.2%) (p=0.256). The history of smoking variable was significantly higher in patients with bladder cancer (88.6-62.5%; p=0.005). BMI was similar in both groups (p=0.345); BMI was 26.5±2.6kg/m^2^(range, 21-32kg/m^2^) in Group-1 and 27.2±4.3kg/m^2^ (range, 21-42kg/m^2^) in Group-2 (p=0.345).

The characteristics of the patients who were diagnosed with bladder cancer are summarized in Table-1.

**Table 1:** Characteristics of the patients with bladder cancer.

	Median	Range	Mean±SD	
**Follow-up duration (month**)	11	9-17	11.6±2.5	
**Size of tumor (mm)**	30	7-80	35.0±17.7	
**Number of tumor**	2	1-10	2.4±2.0	
			n	%
**Tumor stage**	Ta		13	29.5
	T1		31	70.5
**Grade**	Low grade		25	56.8
	High grade		19	43.2
**CIS**	Present		38	86.4
	Not present		6	13.6
**NMP 22**	Negative		22	50.0
	Positive		22	50.0
**Cytology**	Benign		24	54.5
	Malignant		20	44.5
**Recurrence**	No		28	63.7
	Yes		16	36.3
**Progression**	No		41	93.2
	Yes		3	6.8

**Figure 1:**
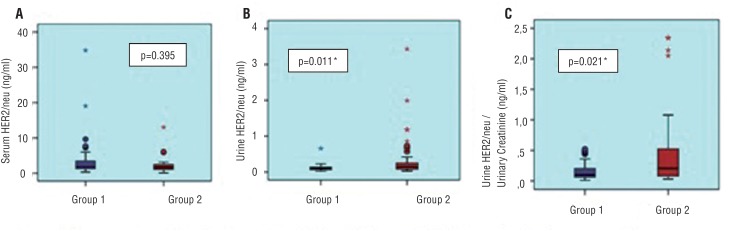
Comparison of patients and controls: a) serum HER2/ner; b) urine HER2/neu levels and c) urine HER2/neu/urinary creatinine ratio.

Between both groups, there was no significant difference in their serum HER2/neu levels (p=0.395) (Figure-1A). The urinary HER2/neu levels and the ratio of the urinary HER2/neu to creatinine were significantly higher in patients with bladder cancer (p=0.011, p=0.021, respectively) (Figures 1B and 1C) (Table-2).

**Table 2:** Comparison of hER2/neu protein levels of patients and controls.

	Control group (n=40)		Bladder cancer group (n=44)		p
	Mean±SD	Range	Mean±SD	Range	
**Urine Her2/neu (ng/mL)**	0.12±0.10	0.0-0.7	0.35±0.61	0.0-3.4	0.011
**Urine Her2/neu / Urine creatinine (ng/mg)**	0.16±0.15	0.0-0.5	0.45±0.62	0.0-2.4	0.021
**Serum Her2/neu (ng/mL)**	3.87±6.08	0.3-34.8	5.84±17.97	0.1-106	0.395

Mann-Whitney U test

The serum HER2/neu level, urinary HER2/ neu level, and urine HER2/neu/urinary creatinine ratio according to smoking status were evaluated. Nevertheless, similar levels were observed both for smokers and non-smokers (p=0.306, p=0.732, p=0.535, respectively). The urinary HER2/neu levels, urinary HER2/neu/creatinine ratio, and serum HER2/neu levels did not show any significant difference between Ta and T1 (p=0.877, p=0.857, p=0.857, respectively). Although no significant difference was observed between high-and low-grade cancer in the urinary and serum HER2/neu levels (p=0.162, p=0.297, respectively), high-grade cancer was found to have a significantly higher urinary HER2/neu/urinary creatinine ratio compared with low-grade cancer (p=0.035). The urinary and serum HER2/neu levels and urinary HER2/neu/urinary creatinine ratio were found to be similar in patients with tumor recurrence and progression and in patients who did not have recurrence and progression. Although there was no relationship between the cytology results and the HER2/neu levels in patients with bladder cancer, those with positive NMP22 were found to have a significantly higher urinary HER2/neu/urinary creatinine ratio (0.55±0.63, 0.35±0.61, p=0.038) and significantly lower serum HER2/neu levels (10.05±24.95ng/mL, 1.63±1.25ng/mL, p=0.015) (Table-3).

**Table 3:** Comparison of HER2/neu levels according to the groups with bladder cancer.

		Urine HER2/ neu (ng/mL)		Urine HER2/neu/urine Creatinine (ng/mg)		Serum HER2/ neu (ng/mL)	
		Mean ± SD	p	Mean ± SD	p	Mean ± SD	p
**Smoking**	smokers	0.19±0.27	0.732	0.34±0.46	0.535	2.95±2.45	0.306
	Non-smokers	0.26±0.51		0.31±0.49		5.51±15.53	
**Tumor stage**	Ta	0.33±0.53	0.877	0.44±0.61	0.857	9.52±28.98	0.857
	T1	0.36±0.65		0.46±0.64		4.30±10.88	
**Grade**	Low	0.27±0.43	0.162	0.31±0.49	**0.035**	8.25±23.63	0.297
	High	0.46±0.78		0.64±0.74		2.67±2.81	
**NMP22**	Negative	0.26±0.44	0.160	0.35±0.63	**0.038**	10.05±24.95	**0.015**
	Positive	0.45±0.74		0.55±0.61		1.63±1.25	
**Cytology**	Benign	0.26±0.41	0.579	0.39±0.60	0.333	8.69±0.60	0.651
	Malignant	0.47±0.78		0.53±0.65		2.43±0.65	
**Recurrence**	No	0.34±0.66	0.634	0.39±0.57	0.420	7.85±22.32	0.575
	Yes	0.38±0.53		0.57±0.71		2.33±2.91	
**Progression**	No	0.37±0.63	0.596	0.47±0.64	0.895	5.86±18.57	0.969
	Yes	0.17±0.03		0.24±0.16		5.55±6.51	

Kruskal-Wallis/Mann-Whitney U test

The HER2/neu levels were not correlated with age, BMI, and the size and number of tumors (Table-4).

**Table 4:** Correlation of hER2/neu levels to age, BMI and the size and number of tumors.

		Age (year)	BMI (kg/m^2^)	Size of Tumor (mm)	Number of Tumor
**Urine HER2/neu**	r	-0.014	-0.022	0.277	-0.057
	p	0.897	0.843	0.068	0.711
**Urinary HER2/neu/ Urinary Creatinine**	r	0.178	0.122	0.108	-0.070
	p	0.105	0.269	0.483	0.649
**Serum HER2/neu**	r	-0.073	-0.102	-0.056	0.172
	p	0.512	0.363	0.722	0.277

Spearman's rank correlation

**Figure 2:**
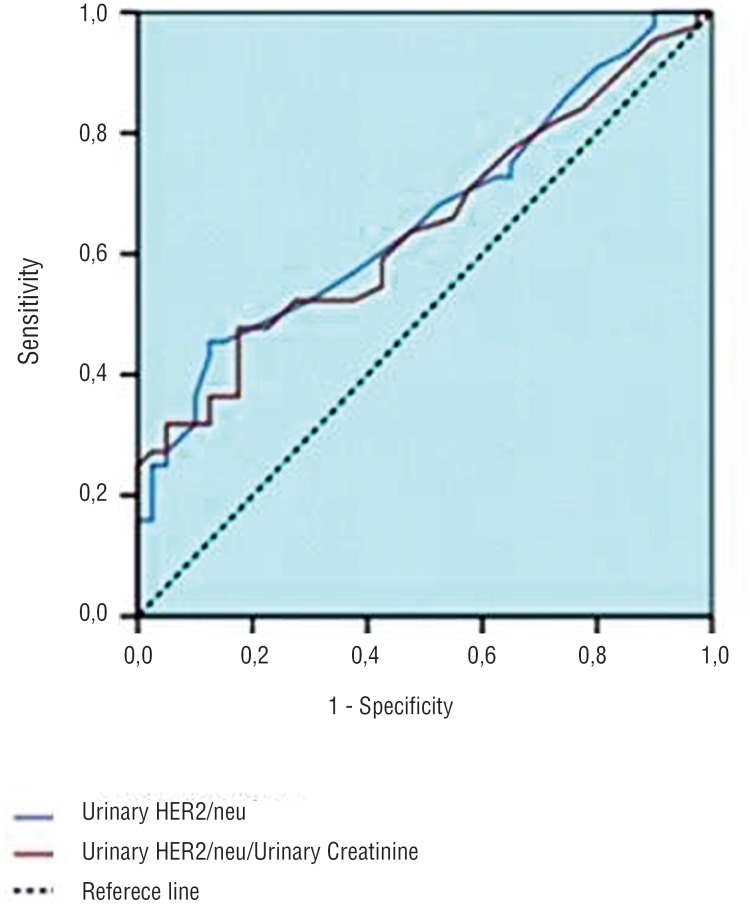
ROC curve for urinary HER2/neu level urinary HER2/neu/creatinine ratio.

Using the cut-off value of 0.4ng/mL for urinary HER2/neu, the sensitivity of the test was found to be 20.5%, and the specificity was 97.5%. Using the cut-off value of 0.4ng/mL for the urinary HER2/neu/urinary creatinine ratio, the sensitivity and specificity of the test were found to be 31.8% and 87.5%, respectively. The positive predictive values were calculated to be 90% and 73.7%, respectively, for the urinary HER2/neu level and urinary HER2/neu/urinary creatinine ratio (Figure-2).

## DISCUSSION

Studies of tumor markers for the diagnosis and follow-up of patients with bladder cancer have been ongoing for years. The ideal tumor marker is expected to be easy to administer and interpret, to be cost-effective, and to have high specificity to avoid false positive results and high sensitivity to avoid undetected tumors (16). However, to date, no alternative tumor marker has been discovered for the diagnosis and follow-up of bladder cancer that can replace cystoscopy and cytology. In this study, although the serum HER2/ neu levels of the patients with NMIBC were noted to be similar to those of the control group, the urinary HER2/neu levels and urinary HER2/neu level/urinary creatinine ratio were significantly higher in patients with bladder cancer. There may be similar results for the serum HER2/neu levels because only patients with NMIBC were included in the study. There is, however, limited information in the literature considering this issue. A study from Korea analyzed a total of 38 patients with bladder cancer, including 12 patients with muscle invasive bladder cancer, and found similar serum HER2/neu levels between the cancer patients and the control group. However, they did not perform a subgroup analysis, which involved only patients with muscle invasive bladder cancer (13).

In the present study, the ELISA method was preferred for measuring the HER2/neu levels. In addition to ELISA, the FISH, IHA and PCR methods can be utilized to determine the HER2/neu level. There is no precise information regarding the superiority of any of these methods. Whereas FISH and IHA methods measure the HER2/neu level in tumor tissue, ELISA is capable of measuring the HER2/neu level in body fluids. There are no available data concerning ELISA, FISH and IHA, evaluated on the resected tissues from the same neoplastic cases. Several studies have indicated over-expression of the HER2/neu protein and gene amplification in bladder cancer. Though over-expression of the HER2/neu protein reaches 81%, gene amplification is found in approximately 60% of the patients with bladder cancer (15). Ecke et al. evaluated the urinary HER2/neu level using the ELISA method and obtained significantly high values in patients with bladder cancer (12). When Kim et al. applied the ELISA method to analyze the HER2/neu levels in serum and urine samples, they did not observe a significant difference in the serum HER2/neu levels; however, they reported a significant difference in the urinary HER2/neu levels. Moreover, the urinary HER2/neu/urinary creatinine ratio was analyzed in this study, and a significantly higher ratio was obtained in the patients with bladder cancer (13).

Although there was no correlation between the urinary and serum HER2/neu levels and the stage and grade of tumors, the HER2/neu/urinary creatinine ratio was observed to be significantly higher in patients with high-grade tumor. Although Lönn et al. reported a correlation between the HER2/neu levels and NMIBC, Kim et al. did not find any relationship between HER2/neu levels and tumor grade (13, 17). In the present study, no correlation was observed between HER2/neu levels and the recurrence and progression of tumor. Skagias et al. used the IHC method to analyze the pathologies of 80 patients diagnosed with bladder cancer, and they identified HER2/neu over-expression in 52% of the patients. In the same study, although the HER2/neu expression was found to be correlated with tumor grade, cancer-specific survival, and overall survival, the HER2/ neu expression did not show any correlation with recurrence (16). When Rink et al. examined the tumor cells circulating in the serum samples of the patients scheduled for radical cystectomy, they observed high HER2/neu levels in 23% of the patients and emphasized the correlation between the high HER2/neu levels observed in recurrence and survival (18).

After examining the specimens using the FISH method, Fleischmann et al. reported that HER2/neu positivity was more frequently observed in patients with lymph node metastasis (19). Kim et al. identified the correlation between positive cytology and high urinary HER2/neu level. However, the urinary HER2/neu level was correlated with the cytological findings in the current study, and the urinary HER2/neu/urinary creatinine ratio was found to be significantly higher in NMP22 positive patients. Similarly, this study did not find any correlation between the HER2/neu levels and the size and number of tumors, and the age and BMI of the patients (13).

The number of smokers was higher in the group of patients with bladder cancer compared with the control group. Although smoking is known to increase the risk of bladder cancer by 3-fold, no relationship was detected between smoking and the HER2/neu levels in this study. Additionally, no HER2/neu mutation was observed in the study of Li et al., who genetically examined a patient group comprising 230 smokers (20).

In two different studies analyzing the urinary HER2/neu levels as potential tumor markers, the sensitivity of the test varied between 71.1% and 88.9%, whereas the specificity varied between 62.5% and 84% (12, 13). Compared with other relevant studies, the sensitivity of the urinary HER2/ neu level was lower and the specificity was higher in the present study. In addition to the serum and urinary HER2/neu levels, the urinary HER2/neu urinary creatinine ratio was analyzed in this study, the findings were considered to be similar to the results relative to the urinary HER2/neu level, which may be attributable to the use of different kits for the tests. There is neither a specified and commonly accepted predictive value for urinary HER2/neu nor an accepted technique for measuring the HER2/neu level. Cytology, a tumor marker with high specificity, has been used for years. Although its specificity exceeds 90%, particularly in patients with high-grade bladder tumor, its sensitivity reaches 60% (16). Because of the high specificity, guidelines recommend that cytology be used along with cystoscopy, especially in the follow-up of high-risk patients. Although the specificity of urinary HER2/neu was observed to be 97.5% in both high and low-grade cancer patients in the present study, considerably low sensitivity was obtained in the HER2/neu tests. Because the specificity of urinary HER2/neu level is high and not dependent on the tumor grade, urinary HER2/ neu may be a new alternative to cytology.

Excluding the studies conducted in search of tumor markers, there are several studies that evaluate the potential therapeutic use of the monoclonal antibody trastuzumab and EGFR-HER2 inhibitor lapatinip in bladder cancer (15, 21). However, there is currently insufficient information in the literature regarding the benefit of these agents for the treatment of bladder cancer. Nevertheless, measuring the HER2/neu levels before and after treatment may be important, as in breast cancer, when these agents are administered to treat bladder cancer. Larger, randomized, controlled trials may shed light on this issue in the near future.

Several limitations of our study should be noted. These limitations include the following: the small sample size, the inclusion of only non-muscle invasive bladder cancer, a single center study, no standard measurement technique of HER2/neu protein and, finally, the short follow-up. The ELISA method and commercial kits were employed to determine the HER2/neu levels. There is no standardization on this subject, and the method used to ensure more accurate results should be identified. Enriching the study with immunohistochemical tissue examinations and genetic methods, in addition to the ELISA method, might have clarified the uncertainty.

## CONCLUSIONS

Although the urinary HER2/neu level and the ratio of urinary HER2/neu level to urinary creatinine level using the ELISA method were significantly higher in patients with NMIBC compared with the healthy patient group, the serum HER2/ neu levels were similar in both groups. Although no correlation was observed between the urinary HER2/neu level and tumor grade, tumor stage, cytology, and NMP22 results, the urinary HER2/ neu/urinary creatinine ratio was found to be correlated with the tumor grade and NMP22 positivity. The urinary HER2/neu test was found to have high specificity but low sensitivity for patients with non-muscle invasive bladder cancer. Additional studies are necessary to better understand the clinical importance of HER2/neu protein in patients with bladder cancer.
